# Dissociating motor learning from recovery in exoskeleton training post-stroke

**DOI:** 10.1186/s12984-018-0428-1

**Published:** 2018-10-05

**Authors:** Nicolas Schweighofer, Chunji Wang, Denis Mottet, Isabelle Laffont, Karima Bakthi, David J. Reinkensmeyer, Olivier Rémy-Néris

**Affiliations:** 1 Biokinesiology and Physical Therapy, University of Southern California, Los Angeles, USA; 2 Neuroscience graduate Program, University of Southern California, Los Angeles, USA; 3 STAPS, Université de Montpellier, Euromov, Montpellier, France; 4 Montpellier University Hospital, Euromov, IFRH, Montpellier University, Montpellier, France; 5 Departments of Mechanical and Aerospace Engineering, Anatomy and Neurobiology, University of California, Irvine, USA; 6 Université de Bretagne Occidentale, Centre hospitalier universitaire, LaTIM-INSERM UMR1101, Brest, France

**Keywords:** Motor learning, Motor adaptation, Motor recovery, Stroke, Neurorehabilitation, Exoskeleton, Rehabilitation robotics, Movement analysis

## Abstract

**Background:**

A large number of robotic or gravity-supporting devices have been developed for rehabilitation of upper extremity post-stroke. Because these devices continuously monitor performance data during training, they could potentially help to develop predictive models of the effects of motor training on recovery. However, during training with such devices, patients must become adept at using the new “tool” of the exoskeleton, including learning the new forces and visuomotor transformations associated with the device. We thus hypothesized that the changes in performance during extensive training with a passive, gravity-supporting, exoskeleton device (the Armeo Spring) will follow an initial fast phase, due to learning to use the device, and a slower phase that corresponds to reduction in overall arm impairment. Of interest was whether these fast and slow processes were related.

**Methods:**

To test the two-process hypothesis, we used mixed-effect exponential models to identify putative fast and slow changes in smoothness of arm movements during 80 arm reaching tests performed during 20 days of exoskeleton training in 53 individuals with post-acute stroke.

**Results:**

In line with our hypothesis, we found that double exponential models better fit the changes in smoothness of arm movements than single exponential models. In contrast, single exponential models better fit the data for a group of young healthy control subjects. In addition, in the stroke group, we showed that smoothness correlated with a measure of impairment (the upper extremity Fugl Meyer score - UEFM) at the end, but not at the beginning, of training. Furthermore, the improvement in movement smoothness due to the slow component, but not to the fast component, strongly correlated with the improvement in the UEFM between the beginning and end of training. There was no correlation between the change of peaks due to the fast process and the changes due to the slow process. Finally, the improvement in smoothness due to the slow, but not the fast, component correlated with the number of days since stroke at the onset of training – i.e. participants who started exoskeleton training sooner after stroke improved their smoothness more.

**Conclusions:**

Our results therefore demonstrate that at least two processes are involved in in performance improvements measured during mechanized training post-stroke. The fast process is consistent with learning to use the exoskeleton, while the slow process independently reflects the reduction in upper extremity impairment.

## Background

Initial behavioral changes post-stroke largely result from “spontaneous recovery”, which is greatest in the first month and continues for ~ 6 months [1]. Spontaneous recovery involves reduction in lesion edema, ischemic penumbra, and brain reorganization [2, 3]. However, motor training consisting of thousands of movements over weeks has been shown to influence the speed and the level of recovery via slow re-organization of surviving neural networks in animals, and is thought to have a similar effect in humans [4–8]. Thus, “recovery” can be thought of as a process of spontaneous recovery modulated by use-dependent plasticity; this recovery is often characterized as a reduction of impairment, measured using clinical scales such as the UEFM scale, which tests the ability to perform a variety of arm and hand movements.

Because therapists can only deliver a fraction of such large doses of training, a number of robotic or mechanized devices have been developed to retrain arm movements post-stroke in a semi-automated manner, see for reviews [9–13]. Upper extremity training with such devices, or even reach training without any mechanical support, has been shown to improve reaching movements’ speed, smoothness, and range (e.g., [14–16]).

During robotic or gravity-supported mechanized training, novel sensorimotor interactions must be learned. For instance, the passive Armeo Spring exoskeleton (Hocoma Inc.), which was used in the present study, applies novel forces to the participant’s arm, because adjustable springs compensate for the impact of gravity on the upper and lower arm. In addition, novel visuo-motor transformations must be learned, because the movements’ goals and hand movements are presented visually on a monitor in front of the participants. To perform fast and accurate movements, participants thus need to learn to compensate for these new forces and visuo-motor transformations. Even after learning to compensate for these perturbations, further performance improvements would be expected with ongoing practice [16, 17].

As a result, the causes of the observed improvements in motor performance shown by individuals post-stroke during exoskeleton training are unclear: Are the improvements due to learning to move the new “tool” of the exoskeleton, or to a reduction of impairment, or both? And, are the two putative processes related? Answering these questions could provide insights into the more general problem of learning new motor tasks after stroke, and even perhaps into the problem of relearning to control the arm with the residual neural hardware that the stroke presumably has re-configured.

Here, our objective was to identify if performance improvements followed two processes during exoskeleton training post-stroke. 53 individuals with moderate impairments due to a stroke between 20 and 90 days prior to enrollment received Armeo Spring training twice a day, every weekday, for four consecutive weeks. Vertical arm reaching tests, performed on the Armeo Spring, were given before and after each session. A group of 11 young healthy control subjects received training for 1 week for comparison of putative motor learning effects.

We used double exponential mixed-effects models to decompose test performance data into faster and slower improvements of performance. Our measure of performance was movement smoothness during pointing tests, given by the average number of peaks in the hand trajectory velocity profiles, a measure that has been previously shown to be sensitive to stroke impairment [14, 15]. The use of an exponential term to model performance gains was motivated by the well-known negatively accelerated gains in performance as a function of training in most motor learning tasks [18], and by recent studies showing that changes in performance in arm reach training in non-disabled and post-stroke individuals can be well modeled with exponentials [19, 20]. The use of the mixed-effects in the nonlinear model was motivated by the high variability in impairment, spontaneous recovery, motor learning, and responsiveness to therapy post-stroke [19, 21, 22]. We compared the fit of double exponentials to single exponentials in the stroke group and in a group of young healthy control subjects. We then tested whether the slow component could assess recovery by comparing changes in smoothness due to the slow component to changes in the Upper Extremity Fugl Meyer (UEFM) pre- and post-training. We also tested whether the fast and slow components correlated with each other, as well as with the start time of the exoskeleton training, relative to the onset of the stroke.

## Methods

### Participants

We analyzed arm kinematic data from a sub-cohort of participants included in the experimental group of the REM-AVC clinical trial (NCT01383512), a multi-center RCT of mechanized arm therapy post-stroke. This RCT aimed at evaluating the medico-economic benefits in post-acute stroke of 4 weeks of standard care and motor arm therapy with Armeo Spring vs. standard care and self-rehabilitation. Kinematic and clinical data of 53 participants with a single stroke in the territory of the middle cerebral artery (MCA) were available for the present study (30 males, 19 females, 4 gender not available; 59.3 ± 13.9 years old; UEFM at baseline 24.7 ± 9.1, days since stroke 56 ± 21 days - all reported values are mean ± standard deviation; see Table 1). The participants were scheduled to receive 4 weeks of Armeo Spring training, 5 days/week, twice/day, for a total of 40 sessions. A performance test was given before and after each training session (thus, for a total of 2 × 2 × 5 × 4 = 80 tests). For each test, we recorded upper limb kinematics during fast and accurate pointing movements (performed with the Armeo Spring) between targets in the vertical plane. Baseline UEFM was measured in the week before training and again in the week following training by physical or occupational therapists who were all trained to administer the UEFM. To quantify normative performance, 11 non-disabled individuals (4 females, 23.5 ± 2.0 years) performed 10 training sessions for 5 days. A performance test was given before and after each training session (thus, for a total of 2 × 2 × 5 = 20 tests).

### The Armeo spring device

**Fig. 1:**
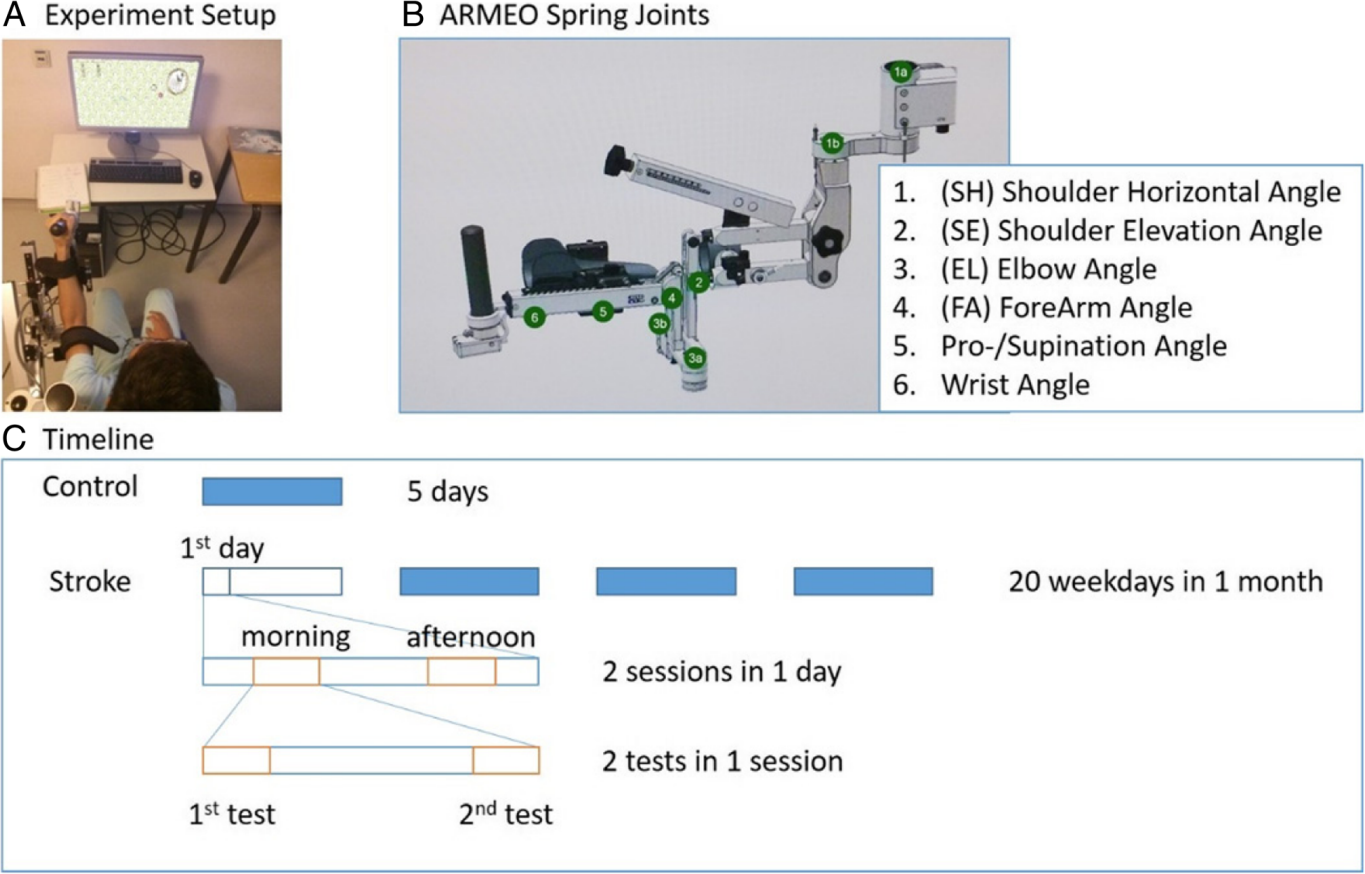
Methods. **a**. Experiment setup: Participants sat in front of a vertical screen on which the video games were displayed. In the “Ladybug Test” given at the beginning and end of each training session, the cursor on the screen represented movements of the end-effector (hand) in the frontal plane as the participant attempted to acquire targets. **b**. The exoskeleton used in this study, the Armeo Spring device. Summation of joints 1a and 1b gives the Shoulder Horizontal (SH) angle, joint 2 the Shoulder Elevation (SE) angle, joint 3a (for the right arm) and 3b (for the left arm) the elbow (EL) angle, joint 4 the ForeArm (FA) angle, joint 5 the pronation and supination angle, and joint 6 the wrist angle. **c**. Training and testing schedule: each day, a session was administered in the morning and a second in the afternoon. The Ladybug test was administered at the beginning and end of each training session. The stroke group received 20 consecutive weekdays of training, whereas the control group received 5 consecutive days of training

The Armeo Spring exoskeleton is based on the T-WREX device [23]. It has six degrees of freedom, summarized in Fig. 1b. It has two adjustable gravity-compensating springs, at the upper arm and the forearm respectively. Lengths of the segments are adjustable to adapt to the user’s arm length. There are no motors at any joints; users must move their arms actively to control the exoskeleton. The arm is attached to the exoskeleton via Velcro straps; movement of the user’s trunk is mildly constrained by a Velcro strap at the upper arm.

**Table 1:** Participants information

Group	No.	Age	Affected Side	Gender	UEFM pre to post	Post Stroke Days	Prescribed No. of Tests
Stroke	53	59 ± 14	29 L, 24R,	30 M, 19F, 4 Missing	25 ± 9 to 39 ± 14 4 Missing	56 ± 21 4 Missing	80 in 4 weeks
Control	11	23 ± 2	–	7 M, 4F	–	–	20 in 1 week

The device records all joint angles and calculates in real time the end effector location through a forward kinematics model of the exoskeleton (developed by Hocoma, Inc.). The end effector location is used to control a cursor on a screen, displayed in the vertical plane in front of the user.

### Exoskeleton training and testing

A training session lasted between 20 and 30 min, and consisted of a performance test (the “Ladybug Test” Fig. 1a) that required moving the cursor to acquire targets shown on the screen, a number of different video games (selected by the therapists and patients in each session), and a second Ladybug test (thus, there were two Ladybug tests/session, and four tests per day). All games and tests, including the Ladybug test, were developed by Hocoma as part of the Armeo Spring software.

The Ladybug test was a two-dimensional pointing task in the frontal plane. In this test, the user was instructed to catch ladybug targets that appeared sequentially on the screen by moving the cursor to the target locations. The movement along the dimension perpendicular to the frontal (coronal) plane was ignored in the control of the cursor. The sequence of target locations appeared random to the subject, but was fixed in each test. The user had limited time to catch the ladybug (< 10 s). After a ladybug was caught, or the time limit was reached, the ladybug disappeared and the next ladybug appeared at a new location. There were four possible difficulty levels for the test; difficulty was modulated both by the number of targets and by the workspace size; from easy to difficult: 12 targets, 24 × 16 cm (horizontal × vertical); 20 targets, 36 × 27 cm; 32 targets, 45 × 36 cm and 48 targets, 63 × 45 cm. In the stroke group, test difficulty was adjusted by the therapist based on the patient’s performance and motivation. In the control group, difficulty was set to the highest level. Here, we only analyzed end-effector trajectory data from these tests, that is, not from the video games in between the tests.

### Data analysis

#### Preprocessing

We filtered the end effector trajectory with a second order Butterworth filter [24] with a cutoff frequency of 5 Hz. We defined a trial as the movement between two consecutive targets. A trial was considered successful if it started from a previously caught target and led to the catching of the next target. Only successful trials, that is, trials in which the participant caught the next ladybug within the pre-specified time of 10 s (control 99.8%, stroke 78.2%) were included in the analysis. We excluded tests in which participants caught less than 25% of the ladybugs (0.7% of all tests in stroke group, 0% in control group).

#### Task space performance

We characterized performance in a test via the average number of peaks in the velocity profile of each successful movements. To calculate the number of peaks in each test, we computed the derivative of velocity (tangential acceleration) and counted the number of times it went from positive to negative. We then took the average of number of peaks (*p*) of all successful trials in the test. Note that the best possible performance is 1 velocity peak per movement.

#### Mixed effect models of learning and recovery

We modeled the dynamics of average number of peaks *p* in the velocity profiles in each test as exponential functions of time *t* represented by test number, with mixed effects [25]. For participants in the control group, visual observation seemed to indicate that changes in the average number of peaks decreased according to a single exponential-like decay. We therefore considered a model with a single exponential formulation, which gives performance for each test as:

1 $$ {p}_{i,j}={A}_i\exp \left(-{B}_i{t}_j\right)+1+{\epsilon}_{i,j}, $$

where *t*_*j*_ is the test number *j*, *A*_*i*_ is the mixed-effect coefficient representing the amplitude of the exponential for participant *i*, *B*_*i*_ is the mixed-effect coefficient representing the learning rate, and *ϵ*_*i*, *j*_ is the residual. We chose an asymptote of 1 because it is the theoretical lower limit of number of peaks in velocity profiles.

For the participants in the stroke group, visual observation seemed to indicate that changes in number of peaks over four weeks of training was initially fast and then slower. We therefore considered a model with two exponential components

2 $$ {p}_{i,j}={A}_i^f\exp \left(-{B}_i^f{t}_j\right)+{A}_i^s\exp \left(-{B}_i^s{t}_j\right)+1+{\epsilon}_{i,j}, $$

where, for all participants, the *A*^*f*^*, A*^*s*^*, B*^*f*^ and *B*^*s*^ were constraint to be positive; in addition,*B*^*f*^ > *B*^*s*^; thus, the first term represents a fast component and the second a slow component. Note that the model of Eq. (1) comprises 5 parameters (the mean and standard deviations of the amplitude and the learning rate, and the residual standard deviation) and the model of Eq. (2) comprises 9 parameters. The mean parameters are the fixed effects and the standard deviation capture the random effects, which model the large variability in lesion, impairment, spontaneous recovery, motor learning, and responsiveness to therapy post-stroke [19, 21, 22].

In model development, it is possible to increase the fit by adding parameters, but doing so may result in overfitting. The Bayesian Information Criterion (BIC) attempts to resolve this problem by introducing a penalty term for the number of parameters in the model. For the control group, the BIC showed that a model with a single exponential better fit the data than a model with two exponentials. We also verified that a model with random effects on both the amplitude and the learning rate better fit the data than a model with only fixed effects. In contrast, for the stroke group, a model with two exponentials better fit the data than a model with a single exponential for this group. We also verified that a model with random effects on the amplitudes and the learning rates for both components better fit the data than a model with only fixed effects. The models were all fit with the function *nlmefit*() in Matlab 2016a. Our predefined threshold for statistical significance was 0.05. All analyses were performed in Matlab.

## Results

**Fig. 2:**
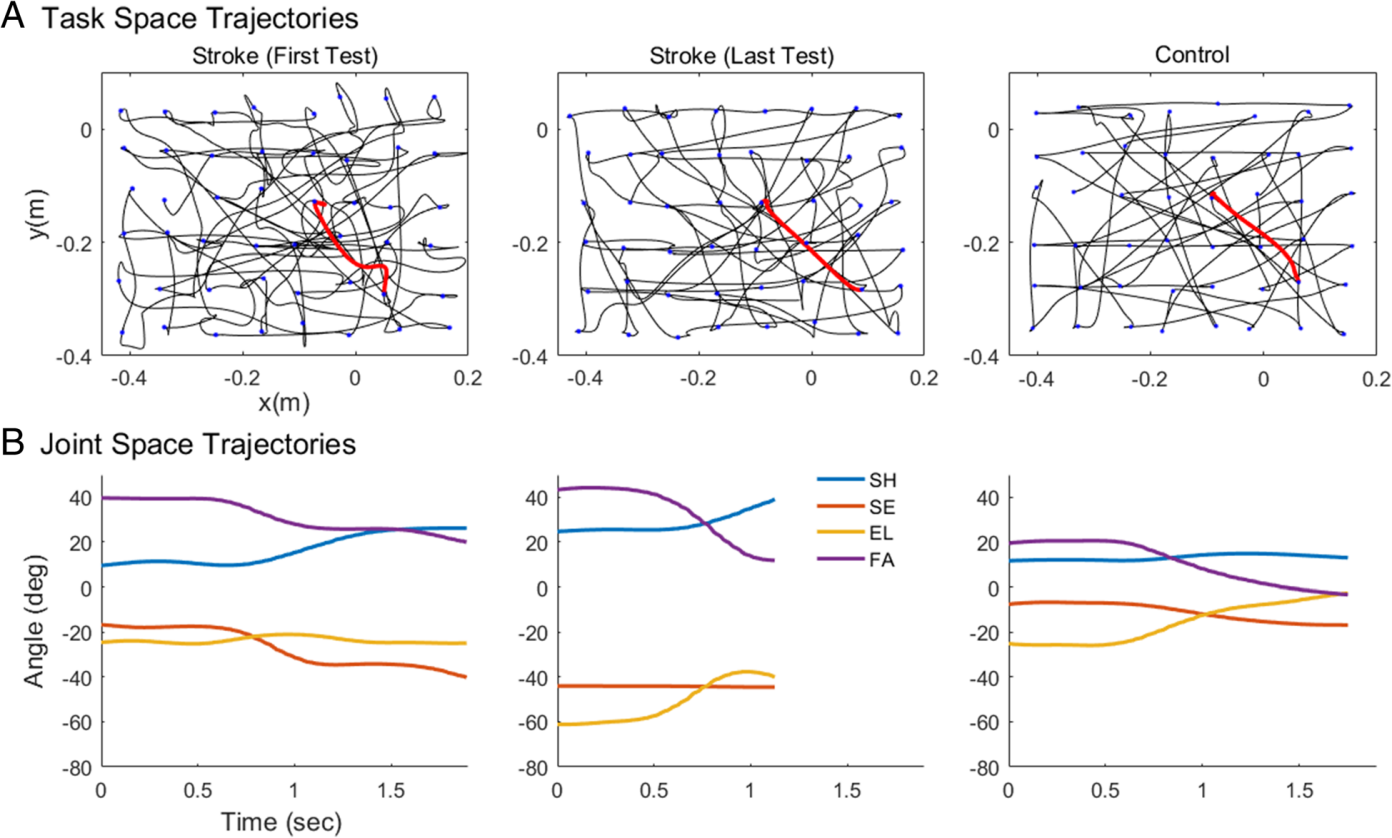
Representative trajectories for an individual in the stroke group in the first and last Ladybug test and for an individual in the control group in the last test. **a**. Representative task space trajectories (units are meters). Note how trajectories after training are straighter for the individual in the stroke group, but still less straight than for the individual in the control group. **b**. Representative joint space time series, correspond to the trajectories shown in red in panel **a**, moving from the center to bottom right. Only the first four exoskeleton joints are shown (units are degrees)

Participants in the stroke group performed an average of 74 ± 13 performance tests, with a range of 33 to 86 tests. 36% of the participants performed 80 tests or more.^1^ All participants in the control group performed the 20 tests. Participants typically showed large changes in performance during the duration of training. Figure 2 shows representative trajectories in both task space and joint space for a participant in the stroke group (UEFM pre = 39, post = 63) and a comparison for a participant in the control group. After training, the trajectories in task space were straighter, and the joint trajectories appeared smoother, but still less so than for the individual in the control group.

**Fig. 3:**
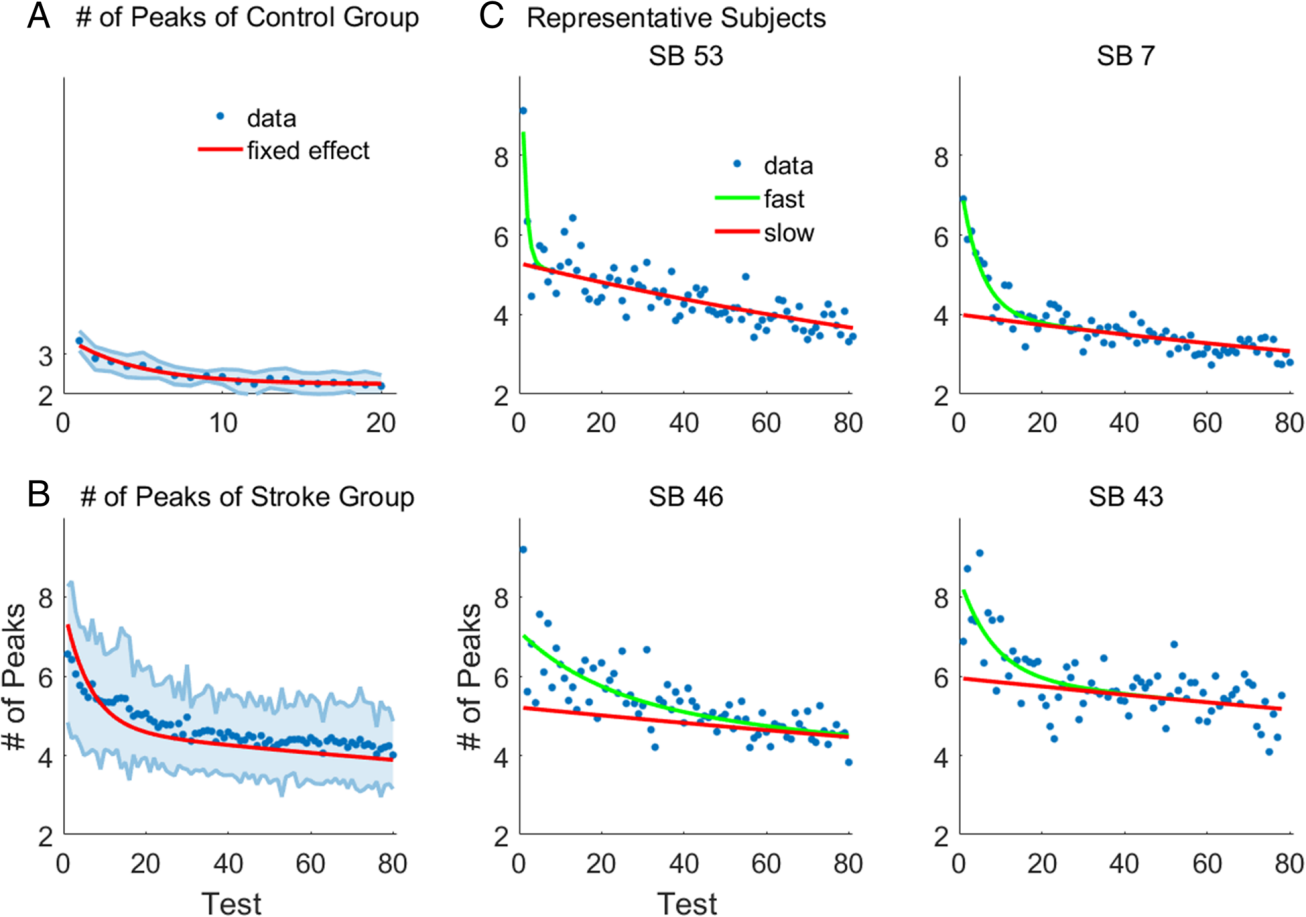
Average number of peaks per trial in velocity profile in ladybug tests: data and mixed effects exponential models fits. Group average (blue dots) and fixed effects (red line) of the fitted mixed-effect exponential models of number of peaks for the control (**a**; single exponential model) and for the stroke group (**b**; double exponential model). Shaded area represents standard deviation. The plots were constructed by using the fixed effects of the mixed effect models. **c**: Representative participants in the stroke group with fast (SB53), slow (SB46) and intermediate (SB 7; SB 43) motor learning rates. The green line represents the summed effects of fast and slow components, and the red line the effect of the slow component for each individual. The plots were constructed by estimating the random effects for each participant and adding the fixed effects

Figure 3 shows the average number of velocity peaks in each trial as well as the models plotted with the fixed effects. The participants in the stroke group exhibited large improvements in the number of peaks, with a decrease of approximately three peaks per movement on average over the course of training (Fig. 3b). Control subjects also showed an improvement in performance, but exhibited fewer number of peaks on average before training, and as a result a smaller overall improvement was possible (Fig. 3a).

Figure 3c shows examples of data and model fits for four individuals post-stroke. The slow component, due to a small learning rate, was approximately linear; thus, the average number of peaks continued to decrease until the end of training. In contrast, the fast component decayed much faster, and usually reached an asymptote during training. For the stroke group, the slow learning rate corresponded to a time constant of 352 ± 123 tests, and the fast learning rate corresponded to a time constant of 13 ± 19 tests. For the control group, the time constant was 4.4 ± 1.6 tests (Note: there were 4 tests per day for both the stroke and control groups, but training and testing was only performed on weekdays, thus we cannot estimate the time constants in days).

**Fig. 4:**
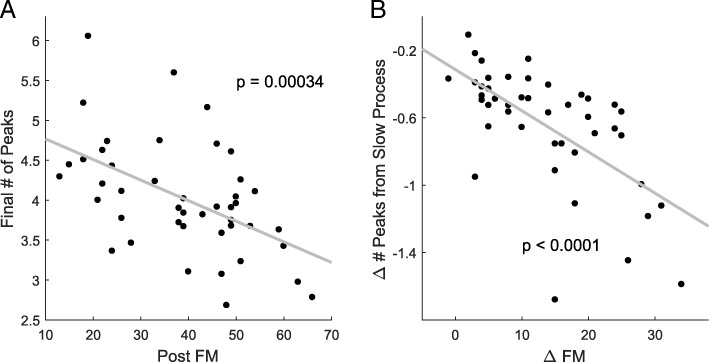
Relationships between movement smoothness and impairment levels. **a**: The estimated number of peaks at the end of training correlates with the UEFM post training. **b**: The estimated change of number of peaks due to the slow process correlates with the change of UEFM, pre to post training. Thus, the slow component corresponds to recovery as measured by the changes of UEFM

For the stroke group, the average number of peaks estimated by the mixed effect model at the last test (test 80) for each participant was significantly correlated with the UEFM post-training (least square regression: *p* = 0.0003; *r* = − 0.52; Fig. 4a). In contrast, the UEFM pre-training did not correlate with the number of peaks at test 1, as estimated by the model (*p* = 0.10). This indicates that the average number of peaks in the velocity profiles as measured by the exoskeleton is only a good indicator of impairment after sufficient practice.

Because we hypothesized that the slow component will measure recovery, we correlated the changes in number of peaks from start to end of training due to the slow component with the changes in UEFM from pre- to post-training. We found that the changes in number of peaks from test 1 to test 80 due to the slow component was significantly correlated with the change of UEFM (both least square and robust regression: *p <* 0*.*0001; *r* = − 0.64; Fig. 4b). In contrast, the changes in number of peaks from before to after training due to the fast component did not correlate with the change of UEFM (*p* = 0.33). In addition, there was no correlation between the change of peaks due to the fast process and the changes due to the slow process (*p* = 0.37).

**Fig. 5:**
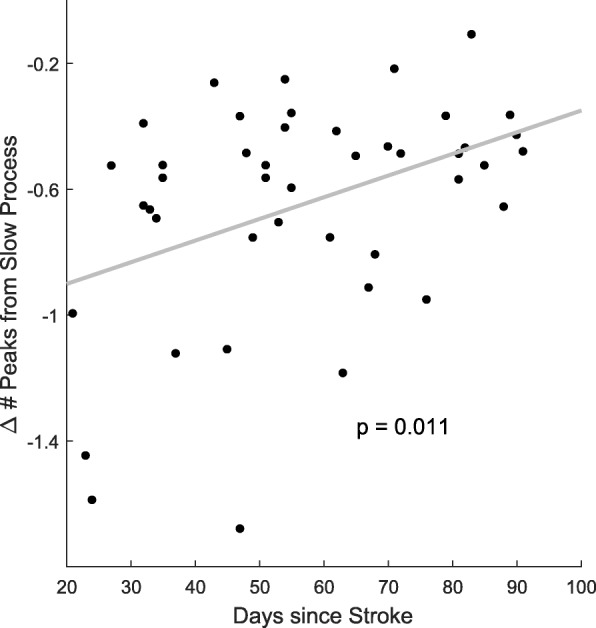
Change in smoothness assessed with the slow component correlates with days since stroke at the onset of training

Finally, we investigated whether the time at which the subjects began the exoskeleton training, relative to the onset stroke, correlated with either motor learning, as estimated via the fast component, or motor recovery, as estimated via the slow component. We found that days since stroke to start of training correlated with the changes in number of peaks from test 1 to test 80 due to the slow component (*p* = 0.0063; Fig. 5): subjects who began training earlier had a greater reduction in peaks. There was no correlation between days since stroke and the changes in number of peaks due to the fast process (*p* = 0.26). Note that we also assessed possible effects of gender, hand affected, and age but found no correlation with changes in the number of peaks due to either fast or slow processes.

## Discussion

We characterized changes in sensorimotor performance in 53 post-stroke individuals during 4 weeks of mechanized motor training with double exponential mixed-effect models. Sensorimotor performance was quantified with the average number of peaks in velocity profiles, an indicator of movement smoothness, in frontal plane reaching tests given before and after each training session. A model that estimated a fast improvement in smoothness via a fast decreasing exponential component, and a slow improvement in smoothness via a slow decreasing exponential component provided a better fit to the data than a model with a single exponential for individual post-stroke. For individuals post-stroke, our results show that the slow, but not the fast component, assessed reduction in upper extremity impairment (i.e. recovery, as we have defined it), because 1) the final average number of peaks as estimated by the model correlated with the post-training UEFM, 2) the changes in number of peaks due to the slow component, but not the overall changes in number of peaks, correlated with the changes in UEFM from pre- to post-training, and 3) the changes in number of peaks due to the slow component, but not the fast component, correlated with the number of days since stroke at the onset of training. The fast component therefore presumably tracked performance improvement due to learning to perform arm movements with the Armeo Spring exoskeleton. Evidence for such motor learning was additionally supported by the fact that non-disabled subjects also showed fast improvements in average number of peaks albeit with a single time constant about three times shorter than that for the fast component of the stroke group. This difference in time constants could be due to age (e.g., [26]) or the effects of stroke, but the single time constant presumably reflects the process of learning to move the exoskeleton, a task the individuals with stroke also had to learn.

There was no correlation between the change of peaks due to the fast process and the changes due to the slow process. This suggests that learning to use the tool of the exoskeleton progresses relatively independently of the ongoing the reduction in upper extremity impairment (c.f., [27]). In other words, there appears to be limited transfer between learning the task of pointing with the exoskeleton, and learning the various movement tasks associated with scoring well on the UEFM.

Early in training in the stroke group, the velocity profiles have multiple peaks and the appearance of a sequence of pulses, suggesting that the pointing task is being executed as a sequence of sub-movements [28]. As previously found in individuals post-stroke [14, 15, 28], these disconnected sub-movements blend to form a trajectory profile with fewer peaks as recovery proceeds. Previous work on a 2D rehabilitation robot showed that such sub-movements exhibited a remarkably invariant speed-vs.-time profile; this was proposed as evidence for dynamic movement primitives, which would constitute fundamental building blocks of complex motion [28]. It is therefore possible that during training, individuals post-stroke relearn incrementally how to (re-)combine multiple primitives for smooth and accurate motor control [29–31].

Our results also show that the rate of motor recovery, but not the rate of motor learning, was higher when subjects started exoskeleton training sooner after their stroke. Animal research has shown that motor training effectiveness depends on time post-stroke, suggesting a critical window of plasticity [3]. In humans, earlier training has been associated with better outcomes [32, 33] although too early intensive training may lead to subpar outcomes [2, 34]. The critical window has been hypothesized to be due to enhanced level of plasticity in the neural milieu near the infarct [3]. Plastic processes involved in force field adaptation or visuo-motor adaptation, as experienced when performing movements with the Armeo Spring, are thought to occur in the cerebellum [35–38]. The participants included did not have cerebellar strokes (since a stroke in the MCA was an inclusion criterion in REM-AVC). Our results thus suggest that the initial cerebellar-dependent motor learning phase does not follow a critical window of plasticity for lesions occurring in the territory of the MCA. In contrast, our findings are overall consistent with a larger rate of spontaneous recovery early after stroke, e.g. [1].

Stroke is characterized by high variability in impairment, spontaneous recovery, and responsiveness to therapy [21]. We have previously shown that including mixed-effects in non-linear models can account for between-individual differences in performance and changes of performances during motor training post-stroke [19]. Here, similarly, we therefore used non-linear mixed effects to precisely account for differences in baseline performance, as well as learning and recovery for each participant. The double exponential model with only nine free fixed parameters (4 means, 4 variances and a noise parameter) fit all data from 53 participants post-stroke simultaneously. Thus, a single mixed-effects model can account for the large between-individual variability in initial and final performance, in learning-related performance changes, and in recovery performance changes. However, although our modeling method identified two time-dependent processes, additional processes are presumably involved, as we have shown with motor adaptation in non-disabled individuals [38]. More sensitive tests that use fewer targets and repeated movements would be needed to identify faster components.

A limitation of our study is the use of arm reaching tests with different difficulty levels set by the therapists for each individual depending on performance. As a results, the number and position of targets varied across and within participants in the stroke group. For this reason, typical metrics used in motor adaptation studies such as maximum deviation from the straight line between the start and target positions could not be used. Hence, our choice to use the average number of peaks in velocity profiles, which does not directly depend on movement amplitudes and is dimensionless. The number of peaks in velocity profiles has long been used to quantify movement smoothness in non-disabled subjects, e.g. [39], and has been previously shown to decrease following robotic training post-stroke [14, 15]). Furthermore, previous studies of arm reaching post-stroke have linked number of peaks in velocity profiles to clinical scores such as the UEFM [40, 41]. Finally, in the present study, the average number of peaks in velocity profiles in each Ladybug test appeared little sensitive to changes in test difficulty, as we verified that that test difficulty was not significant when included as a covariate in the regression analyses. Note however that a robust metric has recently been proposed to measure smoothness in arm movements, the spectral arc-length metric [42]. Nonetheless, these authors concluded that the number of peaks “performs fairly well on the movements made by stroke subjects”, but not on those by non-disabled individuals, presumably because, as we found, the number of peaks are much larger in individuals post-stroke.

Predicting the chances of a patient responding to a specific intervention would be highly useful to clinicians. Because “classical” neurorehabilitation studies generate few repeated measures, typically not during training, the ability to predict recovery based on such data is limited, e.g., [43]. In contrast, studies investigating robotic or mechanized training, such as in the current study, generate large amount of kinematic data that provide a solution to this paucity of repeated data – see [44]. For instance, addition of kinematic measures of arm movements recorded with MIT-Manus after stroke improved prediction of clinical scales of recovery [45]. However, our data show that care must be taken in using initial performance data (extracted from hand trajectories performed with an exoskeleton) as a marker of recovery, because initial performance is not only degraded by the lesioned motor system, but depends on learning to move the exoskeleton.

## Conclusion

Rehabilitation after brain damage is based on the premise that motor learning determines activity-dependent motor recovery after stroke [46]. In this study, kinematic analysis of pointing movements performed during four weeks of motor training with an exoskeleton post-stroke allowed us to identify a fast and a slow process of performance improvement. Because of the fast learning process, changes in kinematic performance early in training were poor predictors of impairment reduction. The current work could not assess, however, whether long-term training with the exoskeleton benefits the patients, as our analyses cannot show a causal effect of motor training on recovery. Such causal effects need to be assessed via clinical trials like REM-AVC or via longitudinal studies in which the dose of training is manipulated as the variable of interest.

## Footnotes

1 Some participants did not perform all the sessions for reasons such as fatigue (the session was terminated before the 2nd test was given) or omission by the therapists to include the test in the session. In addition, some therapists did not include the test in the first sessions when patients were instructed on the functioning of the ARMEO.
