# Correction to: Dissociating motor learning from recovery in exoskeleton training post-stroke

**DOI:** 10.1186/s12984-018-0473-9

**Published:** 2018-12-17

**Authors:** Nicolas Schweighofer, Chunji Wang, Denis Mottet, Isabelle Laffont, Karima Bakhti, David J. Reinkensmeyer, Olivier Rémy-Néris

**Affiliations:** 1 Biokinesiology and Physical Therapy, University of Southern California, Los Angeles, USA; 2 Neuroscience graduate Program, University of Southern California, Los Angeles, USA; 3 STAPS, Université de Montpellier, Euromov, Montpellier, France; 4 Montpellier University Hospital, Euromov, IFRH, Montpellier University, Montpellier, France; 5 Departments of Mechanical and Aerospace Engineering, Anatomy and Neurobiology, University of California, Irvine, USA; 6 Université de Bretagne Occidentale, Centre hospitalier universitaire, LaTIM-INSERM UMR1101, Brest, France

**Correction to: J Neuroeng Rehabil**

**https://doi.org/10.1186/s12984-018-0428-1**

The original article [1] contained an error whereby the co-author, Karima Bakhti’s name was displayed incorrectly. This error has now been corrected.
